# Cuidados de larga duración en Costa Rica: enseñanzas para América Latina desde la evidencia internacional

**DOI:** 10.26633/RPSP.2021.146

**Published:** 2021-11-19

**Authors:** Alexander Chaverri-Carvajal, Mauricio Matus-López

**Affiliations:** 1 Universidad Pablo de Olavide Sevilla España Universidad Pablo de Olavide, Sevilla, España.

**Keywords:** Cuidados a largo plazo, dependencia funcional, protección social, América Latina, Américas, Long-term care, functional status, public policy, America, Latin America, Assistência de longa duração, estado funcional, política pública, Américas, América Latina

## Abstract

La creciente prevalencia de dependencia funcional derivada del envejecimiento acelerado y la transformación epidemiológica hace inevitable la implementación de nuevos sistemas de cuidados de larga duración (CLD) en las Américas. En marzo del 2021, Costa Rica se convirtió en el único país de ingresos medios en la Región que ha iniciado la aplicación de un sistema nacional de este tipo. En este artículo se compara el diseño del nuevo sistema de cuidados de larga duración de Costa Rica, con los sistemas de Australia, Dinamarca, España, Estados Unidos de América, Japón y Uruguay, y se identifican enseñanzas útiles para el desarrollo de otros sistemas de CLD en la Región. Se analizan cuatro aspectos: el marco legal, el acceso y la cobertura, los tipos de servicios, y los costos y la financiación. Para ello se realizó una revisión de la bibliografía científica y de informes nacionales e internacionales entre el 1 de enero del año 2000 y el primer día de abril del año 2021. El incipiente modelo sigue las principales tendencias de la experiencia internacional. Es progresivamente universal, prioriza la atención domiciliaria, incluye herramientas tecnológicas, crea parámetros de calidad para los servicios, incorpora transferencias monetarias para familiares que se desempeñan como cuidadores, inicia servicios de respiro y desarrolla formación para personas cuidadoras. No obstante, la evidencia internacional muestra que los modelos con menor financiamiento tienen baja cobertura y poca diversidad en servicios. La escasa generosidad fiscal, la ausencia de nuevas fuentes de recursos económicos y la debilidad jurídica arriesgan la ampliación, desarrollo y sostenibilidad del nuevo modelo en las Américas.

## Abstract (en)

The growing prevalence of functional dependency as a result of accelerated aging and epidemiological transformation has created a pressing need to implement new systems to address the problem of long-term care (LTC) in the Region of the Americas. In March 2021, Costa Rica became the only middle-income country in the Region that has taken steps to introduce a national LTC system. The present article compares the design of this new LTC system with existing systems in Australia, Denmark, Japan, Spain, the United States of America, and Uruguay, and identifies useful lessons for the development of LTC systems in other countries of the Region. Four aspects are analyzed: the legal framework, access and coverage, types of services, and costs and financing. A search of the scientific literature and national and international reports was conducted between 1 January 2000 and 1 April 2021. The results showed that the incipient Costa Rican model follows the main trends that are seen internationally. It is progressively universal, prioritizes home care, incorporates technology tools, creates parameters for measuring the quality of services, provides monetary transfers for family members who serve as caregivers, includes respite services, and offers training for caregivers. At the same time, however, the international evidence shows that models with poor levels of funding have low coverage and offer little diversity in terms of the services provided. Insufficient funding, absence of new economic resources, and weak legal foundations are jeopardizing the expansion, development, and sustainability of the new model in the Americas.

## RESUMO

A crescente prevalência de dependência funcional derivada do envelhecimento acelerado e da transformação epidemiológica torna inevitável a implementação de novos sistemas de cuidados de longa duração (CLD) na Região das Américas. Em março de 2021, a Costa Rica se transformou no único país de renda média na Região que iniciou a aplicação de um sistema nacional desse tipo. Neste artigo, compara-se o desenho do novo sistema de cuidados de longa duração da Costa Rica com os sistemas da Austrália, Dinamarca, Espanha, Estados Unidos da América, Japão e Uruguai, e se identificam aprendizados úteis para o desenvolvimento de outros sistemas de CLD na Região. São analisados quatro aspectos: o marco legal, o acesso e a cobertura, os tipos de serviço, os custos e o financiamento. Para isso, foi realizada uma revisão da bibliografia científica e de relatórios nacionais e internacionais entre 1º de janeiro de 2000 e 1º de abril de 2021. O modelo inicial segue as principais tendências da experiência internacional. É progressivamente universal, prioriza a atenção domiciliar, inclui ferramentas tecnológicas, cria parâmetros de qualidade para os serviços, incorpora transferências monetárias para familiares que atuam como cuidadores, inicia serviços de cuidados intermitentes e desenvolve formação para cuidadores. No entanto, a evidência internacional mostra que os modelos com menor financiamento têm baixa cobertura e pouca diversidade de serviços. A escassa generosidade fiscal, a ausência de novas fontes de recursos econômicos e a vulnerabilidade jurídica colocam em risco a ampliação, o desenvolvimento e a sustentabilidade do novo modelo nas Américas.

En 1990, la población adulta mayor en el mundo constituía 6% del total; en el 2050 esta proporción se triplicará. Este cambio demográfico genera presiones sobre la demanda de servicios sociales y sanitarios, principalmente en países de ingresos medios y bajos (1,2).

América Latina es el subcontinente que envejece más rápido. Lo hace con bajos niveles de ingresos (es la región más desigual del mundo) y en condiciones poco saludables.

El desarrollo regional de sistemas de cuidados de larga duración (CLD) es escaso. La tendencia ha consistido en la participación limitada del Estado, que se focaliza en personas con vulnerabilidad social y económica. Hasta ahora, la única excepción fue Uruguay, que en 2015 inauguró el primer programa de CLD en América Latina (3). En el 2018, la Comisión Económica para América Latina y el Caribe hizo un llamamiento urgente a ajustar los sistemas de protección social, según perfiles demográficos previstos (4).

Costa Rica es el país latinoamericano con mayor esperanza de vida al nacer. Además, en comparación con la mayoría de los países de la Región, su población envejece con más velocidad. Entre los años 2000 y 2020, la cifra de personas mayores prácticamente se duplicó, y para el 2050, constituirán 24% de la población. En marzo del 2021, Costa Rica se transformó en el segundo país de la Región en comenzar con su programa de CLD, y el primero de ingresos medianos en iniciar este proceso que recorrerán otros países de las Américas. El objetivo de este artículo es identificar enseñanzas útiles para el desarrollo de otros CLD en la Región. Para ello, se compara el diseño del nuevo sistema costarricense con los ejes analíticos de los sistemas de Australia, Dinamarca, España, Estados Unidos de América, Japón y Uruguay.

## MATERIALES Y MÉTODOS

El presente estudio es una revisión exhaustiva documental y bibliográfica de los últimos veinte años, para identificar enseñanzas para aplicar en otros programas de CLD en las Américas mediante la comparación de ejes analíticos de diseño en modelos heterogéneos alrededor del mundo.

### Criterios de elegibilidad y detalles sobre la recopilación de datos

El primer criterio son los ejes de análisis para identificar las enseñanzas. Estos ejes, definidos en la búsqueda inicial, fueron elegidos según su relevancia en la bibliografía internacional y son los siguientes: el marco legal, el acceso y la cobertura, los tipos de servicios, y los costos y la financiación (5-7).

El segundo criterio es la selección de modelos de CLD en todo el mundo para comparar los ejes analíticos. La selección tiene tres variables: que sean sistemas públicos consolidados, que respondan a poblaciones envejecidas y que incluyan distintos continentes. Los criterios de inclusión son: a) países cuyos sistemas públicos de CLD tengan más de cinco años de antigüedad; b) países con 15% o más de población mayor de 65 años; y c) al menos un país por continente y no más de dos.

### Modelos incluidos

La revisión de la bibliografía identificó 36 países con programas de CLD. De ellos, existe certeza de 27 sistemas nacionales. En 22 de estos países, la población adulta mayor supera 15% del total y sus sistemas tienen al menos un quinquenio de existencia. De esta veintena, se seleccionaron los casos disponibles más consolidados y relevantes en la bibliografía internacional para cada continente: de Asia, Japón; de Oceanía, Australia; y de América del Norte, Canadá y Estados Unidos. Se eligió este último por su peso en la bibliografía internacional. En América Latina se eligió el único caso existente. En Europa, se seleccionó uno del norte y otro del sur: el modelo danés, de los más antiguos y desarrollados, y el español, más reciente y menos abarcador, respectivamente. En el continente africano, solo Sudáfrica tiene avances en CLD, pero no cumple los criterios de inclusión.

### Fuentes de información

**FIGURA 1.**
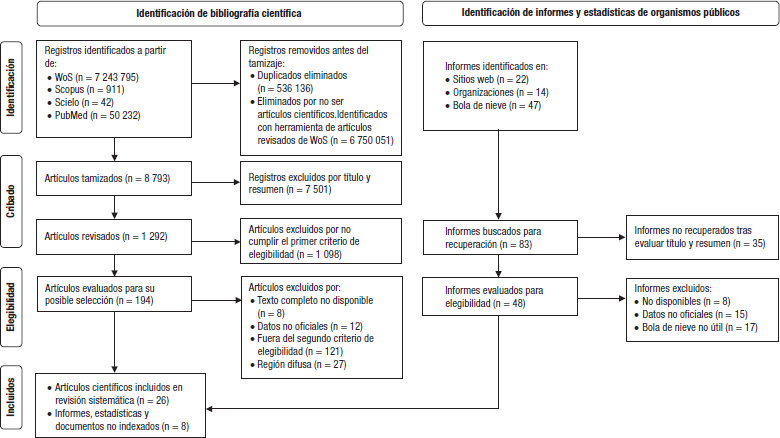
Diagrama de flujo

Se usaron tres fuentes (figura 1). En primer lugar, una revisión de los artículos científicos publicados en Web of Science, Scopus, Scielo y PubMed en el período 2000-2021, que coincide con los nuevos desarrollos y reformas recientes. Los términos de búsqueda (en inglés y español) fueron: [(long-term care OR dependency care) AND (policy OR system) AND (Australia OR United States OR Denmark OR Spain OR Japan OR Uruguay OR Costa Rica)]. En segundo lugar, se realizó una revisión con estrategia de bola de nieve sobre las referencias de artículos, libros y documentos citados en los resultados anteriores y no indexados en las bases científicas. Por último, se realizó una revisión exhaustiva de informes, documentos y estadísticas disponibles en organismos públicos responsables de los programas de CLD en cada país.

***Fuente:*** elaboración propia.

WoS, Web of Science.

**CUADRO 1.** Marco legal y caracterización socioeconómica de programas de cuidados de larga duración en todo el mundo, 2021

**Países**	**Costa Rica**	**Uruguay**	**Dinamarca**	**Japón**	**España**	**Estados Unidos**	**Australia**
Población total	5,1 millones	3,47 millones	5,8 millones	126,5 millones	46,7 millones	331 millones	25,5 millones
Porcentaje de personas de 65 años y más (%)	10,3	15,1	20,2	28,4	20	16,6	16,2
Esperanza de vida (años)	80,4	77,7	80,7	84,4	83,4	78,8	83,2
Ingreso per cápita^a^	$12 244	$16 190	$60 170	$40 247	$29 600	$65 298	$55 060
Ubicación en la lista del IDH	62º	55º	10º	19º	25º	17º	8º
Marco legal (año de creación)	Decreto Ejecutivo 42878-MP-MDHIS (2021)	Ley de creación del SNIC (2015)	Ley de Servicios Sociales (1974)	Ley del Seguro de Dependencia (1997)	Ley de creación del SAAD (2006)	Older Americans Act (1965)	Aged Care Act (1997)

a Ingreso promedio en dólares estadounidenses que cada año recibe cada uno de los habitantes de un país.

IDH, Índice de Desarrollo Humano; SNIC, Sistema Nacional Integrado de Cuidados; SAAD, Sistema para la Autonomía y Atención a la Dependencia.

***Fuente:*** Preparado por los autores con la bibliografía citada.

### Evaluación del riesgo de sesgo

Los criterios para evitar el riesgo de sesgo en la selección de bibliografía fueron: a) revisión del título y resumen; y b) cuando fuera difuso, se revisó el texto completo. Se excluyeron las publicaciones duplicadas, los documentos con datos no oficiales, las fechas fuera del rango y estudios sin delimitación geográfica precisa.

## RESULTADOS

### Marco legal

El Sistema de Apoyo a los Cuidados y Atención a la Dependencia (SNC) costarricense, creado mediante el Decreto Ejecutivo 42878-MP-MDHIS el 3 de marzo del 2021, tiene como objetivo “la implantación progresiva de un sistema de promoción de la autonomía, de apoyo a los cuidados y de atención a la población en situación de dependencia” (8).

El decreto ordena su aplicación desde su promulgación. Este año, se creará una Secretaría Nacional de Cuidados, encargada de coordinar el sistema, y se construirá la herramienta de evaluación de la dependencia para las solicitudes. A fines del 2021, se desplegarán los primeros servicios y, en los años siguientes, la totalidad. En el cuadro 1, además del marco legal que crea los sistemas, se incluye una caracterización socioeconómica de los países comparados.

### Criterios de acceso y cobertura

La cobertura estimada será de 55,9% de la demanda total de personas dependientes entre 2021 y 2031. Esto equivale a 20% de las personas mayores de 65 años y más.

Se determina el acceso universal para adultos dependientes. Estos se definen como las personas que requieren ayuda de otras para realizar actividades de la vida diaria de manera prolongada. Los servicios se prestan según el grado de dependencia del individuo, que puede ser leve, moderado y grave. También incluye a los cuidadores como beneficiarios del sistema (8).

El SNC declara el acceso universal, similar a Dinamarca y España (9,10). Esto no ocurre en otros casos, como Australia, Japón y Uruguay (11-13). El caso de Australia es particular. En términos generales, son beneficiarias todas las personas de 65 y más años. Sin embargo, tiene dos excepciones: a) pueden acceder a partir de los 50 años quienes sufren envejecimiento prematuro y sinhogarismo, y las personas dependientes que pertenezcan a los pueblos originarios y habitantes de las Islas del Estrecho de Torres; y b) pueden acceder desde los 45 años quienes cumplen ambos requisitos a la vez (11).

Al igual que los modelos universales, Costa Rica evaluará la dependencia de las personas, antes de conceder los servicios. Esta medición es similar a la realizada en España, donde se evalúa a los solicitantes con un baremo que clasifica a los dependientes en tres grados. Así, se determina el programa individual de atención, que define los beneficios a los que tiene derecho.

La cobertura esperada del SNC es similar a la media de los países fuera de América (cuadro 2). El sistema uruguayo incluye en su población objetivo a los niños; de hecho, son prioridad. En el 2017 iniciaron los servicios de CLD para personas mayores. Si bien se define universalista, en la práctica, y por motivos financieros, solo conceden servicios de asistente personal a personas de 80 y más años, y teleasistencia desde 70 años. Tras el último cambio de gobierno no hay nuevas estadísticas, y se esperan aumentos en montantes por copago (14).

### Tipos de servicios

Se incluye la prestación de cuatro tipos de servicios: asistencia domiciliaria, atención residencial, centros de día y teleasistencia. La oferta individual se establecerá luego de aplicar el baremo.

#### Orientación de los servicios.

El SNC está orientado a la atención domiciliaria. Incluye hasta 80 horas mensuales y busca cubrir 80% de las personas dependientes graves antes del 2031. Por otra parte, se estima que cerca de 10% de esta población vive actualmente en residencias. Se acreditará la calidad de estos centros y se aumentarán las plazas hasta cubrir 20% de los dependientes graves.

Entre los servicios de atención domiciliaria, la teleasistencia consiste en el contacto telemático entre la persona dependiente y un centro de atención. Se espera una cobertura de todos los dependientes graves y de 70% de los moderados. Los centros de día están dirigidos a dependientes moderados y graves y se espera cubrir al 10% de esta población.

**CUADRO 2.** Comparación de acceso y cobertura de beneficiarios de cuidados de larga duración de Costa Rica y otros países

**País**	**Acceso Universal (Sí/No)**	**Elegibilidad (por edad)**	**Instrumento Nacional de Medición de la Dependencia (Sí/No)**	**Beneficiarios (% de 65 años y más)**
Costa Rica	Sí	Mayor de 18^a^	Sí	˜ 20
Uruguay	Sí	Mayor de 65	Sí	<1
Dinamarca	Sí	Todas las edades	Sí	25,82
Japón	Sí	Mayor de 40	Sí	16,37
España	Sí	Todas las edades	Sí	8,7
Estados Unidos de América	No	Todas las edades	No	10
Australia	Sí	Mayor de 65 (con excepciones)	Sí	31,5

a Las transferencias monetarias incluyen a menores de edad.

***Fuente:*** elaboración propia.

En la actualidad, los servicios provistos directamente por el Estado son escasos. Son gestionados, en su mayoría, por organizaciones sin lucro que operan con transferencias públicas para atender personas en pobreza. El SNC incorpora copago para ampliar la oferta de servicios a personas no pobres.

La evidencia internacional muestra que los sistemas se enfocan principalmente en la atención domiciliaria. En Australia, 77% de los usuarios tienen algunos de los beneficios de apoyos o cuidados domiciliarios (15). Además, las residencias de larga estancia son para los mayores de 80 años (16). El programa de CLD danés es de los más abarcadores en la atención domiciliaria (16). Los beneficiarios de servicios domiciliarios triplican a los de residencias de larga estancia, que dejaron de construirse en 1987 (17).

En Japón, la proporción de servicios domiciliarios es algo menor que en Australia, y representa poco más de la mitad del total. Sin embargo, es líder en cobertura en servicios de base comunitaria, que alcanza a 15,1% de los usuarios (18).

Estados Unidos ha dado un giro reciente hacia los servicios no residenciales. Hasta el 2012, estos eran los más importantes (19). No obstante, debido que algunos estados han adoptado la Ley del Cuidado de Salud a bajo Precio (ACA, por su sigla en inglés) que amplió el programa Medicaid para dar cobertura de servicios domiciliarios y comunitarios a más adultos de bajos recursos y subvencionó las pólizas médicas privadas, en los últimos años incrementó la cobertura a nivel nacional (20). Aun así, en promedio, es el país con más personas en residencias de larga estancia del estudio (21).

En Uruguay, 58,7% de los beneficiarios reciben asistencia personal por un máximo de 80 horas mensuales (22), y en España, 28,5% de usuarios reciben ayuda en su domicilio o el importe para abonar estos servicios, con un máximo de 70 horas mensuales. En ambos modelos se destaca la extensión del servicio de teleasistencia, que cubre a 18,32% de los beneficiarios en España y 35,9% en Uruguay (10,22).

La característica principal del modelo uruguayo es que no incluye la atención en residencias. Existe un programa que podría considerarse parte de estos servicios, gestionado por el Banco de Previsión Social (BPS), aunque está planteado como una solución habitacional y no como un programa CLD. No existe explicación aparente para esta decisión más que la existencia de tensiones institucionales (14).

El modelo español se destaca por estar dividido entre servicios y transferencias. Mediante los primeros están cubiertos el 57,36% de usuarios. Por otra parte, 31,46% de beneficiarios recibe cuidados por medio de transferencias monetarias a hogares (cuadro 3), en general a cargo de familiares no profesionales. Esta última es la más extendida de las prestaciones y servicios del Sistema para la Autonomía y Atención a la Dependencia (SAAD).

#### Transferencias monetarias.

El SNC incluye transferencias monetarias para quienes cumplen uno de estos requisitos: i) que la persona dependiente califique para recibir servicio de asistencia en domicilio, ii) que la persona cuidadora no tenga opciones de incorporarse al mercado laboral, y iii) que se encuentre en situación de pobreza extrema.

En comparación con la evidencia internacional, Costa Rica ha tomado algunas previsiones. España saturó el sistema de transferencias monetarias y ha comenzado a priorizar la concesión de más servicios (10). En otros modelos, estos servicios no suelen ser medulares, sino complementarios a seguros de salud o trabajo, y son temporales. Por ejemplo, en Japón, se pagan hasta 93 días de excedencia a trabajadores que cuiden familiares (23). Algo similar se hace en Dinamarca, donde las personas que cuidan de un pariente cercano o amigo con enfermedad terminal y desea permanecer en su casa, tienen derecho a recibir este pago (cuadro 3). No obstante, solo 0,9% de usuarios del programa de CLD danés optan por esta prestación (24).

#### Personas cuidadoras.

El SNC está elaborando programas de formación para cuidadores, procurando que accedan a empleos formales. Para ello, se creará una certificación oficial de cuidador domiciliario luego de asistir a la capacitación, que dura 810 horas. Además, para cuidadores informales, habrá cursos sobre autocuidado y servicios de respiro familiar, como atención domiciliaria y breves estancias residenciales.

En comparación con la evidencia internacional existente sobre capacitación para cuidadores formales, Japón se destaca porque todos los cuidadores formales realizan cursos, que duran de uno a tres años, y deben aprobar un examen de previo a la inserción laboral. Además, se otorgan incentivos financieros a los proveedores que brindan capacitación continua a sus trabajadores. Los currículos de los programas de formación se revisan cada tres años.

Uruguay tiene un programa nacional de formación para asistentes personales, que dura 158 horas, y que deben cursar todas aquellas personas que quieran recibir recursos del sistema para desempeñarse en esta actividad (13). Por su parte, Estados Unidos no tiene programa federal de formación; sin embargo, en algunos estados se imparten cursos prácticos de dos semanas y clases de entre 75 y 120 horas, según la normativa de cada estado. La capacitación dura alrededor de cuatro meses.

**CUADRO 3.** Enfoques de servicios, prestatarios y beneficios para personas que brindan cuidados de larga duración en Costa Rica y otros países del mundo

**País**	**Servicios de CLD**	**Provisión**	**Transferencias monetarias**	**Personas cuidadoras**
Costa Rica	Orientación de atención en domicilio	Principalmente privado	Excepcional (en situaciones de pobreza)	Capacitación y respiro familiar
Uruguay	Orientación de atención en domicilio	Privado	-	Capacitación
Dinamarca	Orientación de atención en domicilio	Principalmente público	Excepcional (atención familiar)	Capacitación y respiro familiar
Japón	Orientación de atención en domicilio y comunitaria	La mayoría de los proveedores de atención domiciliaria son privados. La mayoría de los servicios residenciales y comunitarios son públicos o sin lucro	Excepcional (atención familiar)	Capacitación y respiro familiar
España	Orientación de atención en domicilio	Principalmente privado	Extensivo	Capacitación
Estados Unidos de América	Orientación de atención en domicilio y comunitaria	Principalmente privado	Excepcional (en algunos Estados)	Capacitación (en algunos Estados)
Australia	Orientación de atención en domicilio	Principalmente privado	Excepcional (atención familiar)	Capacitación y respiro familiar

CLD, cuidados de larga duración.

***Fuente:*** elaboración propia.

Australia se destaca por su oferta variada de servicios de respiro para cuidadores informales. Estos servicios son de cuatro tipos: relevo diurno o nocturno en el domicilio, estancias nocturnas en algún centro comunitario, tiempo en centros de día y estancias más prolongadas en residencias. Se ofrecen por períodos limitados. Se pueden solicitar de manera programada o en una situación de emergencia, con un máximo de 63 días al año, prorrogables. Son utilizados por 9,1% de las personas usuarias, pero de forma complementaria con otros servicios (25).

En Dinamarca, las autoridades locales ofrecen servicios de respiro (cuadro 3), a través de atención domiciliaria o estancia breve en residencias (9).

#### Otros servicios previos a la dependencia.

Los modelos de Dinamarca y Japón han avanzado en servicios de detección de fragilidad y síndromes geriátricos para prevenir el avance de la dependencia y ajustar los servicios. En Dinamarca, los municipios realizan visitas semestrales obligatorias a personas de 75 años y más, y desde los 65 años a aquellos con necesidades adicionales porque, por ejemplo, han perdido a su cónyuge, están aisladas o han recibido el alta hospitalaria recientemente (26).

En Japón, los cuidados preventivos valoran los aspectos físicos y psicosociales relacionados con hábitos de vida saludable como ejercicios, peso y consumo de alcohol y tabaco. Promueven conductas y estilos de vida saludables. Incluyen campañas publicitarias, programas de ejercicios comunitarios y otros como psicoterapia para mejorar funciones cognitivas (27). España tiene servicios de prevención, pero su despliegue es menor, llega al 4,17% de usuarios (10). Costa Rica no incluye servicios de este tipo.

#### Coordinación entre los programas de CLD y los programas de salud.

El SNC incluirá, de manera progresiva, una herramienta de telesalud para que las personas dependientes que no pueden desplazarse al centro médico realicen consultas de manera telemática. Además, los sistemas de CLD y de salud compartirán los datos de los beneficiarios.

Desde un punto de vista comparativo, hay dos modelos que son los más avanzados. Japón inició su plan de promoción de la reforma sociosanitaria de datos, que integrará, de manera electrónica, los registros de los sistemas de salud y de CLD vinculados a nivel nacional (28). Por otra parte, Dinamarca está desarrollando programas de telemedicina que actualizan la base de datos sociosanitarios (26).

### Costos y esquemas de financiación

Se estima que el nuevo sistema costarricense tendrá un costo anual de $235 millones (USD), alrededor de 0,8% del producto interno bruto (PIB). La inversión inicial representa 43,8% del total y se espera completar el presupuesto en diez años. El sistema se financia con impuestos generales y copagos. No considera la creación de impuestos nuevos ni el aumento de los actuales. La inversión inicial se financiará con reasignaciones presupuestarias. Las tasas de copago aún no se han definido (8).

En comparación con la evidencia internacional (cuadro 4), el esquema financiero del SNC es similar al uruguayo, cuyas únicas fuentes son impuestos generales y copagos (14). Este sistema aspira a un aporte similar al del SAAD, el cual se financia con impuestos generales de la administración central y de los gobiernos regionales, y con copagos por renta, patrimonio y tipo de servicio. La composición por fuentes es heterogénea entre los territorios, en función del aporte de cada gobierno regional y los copagos recaudados (29).

El sistema público en Estados Unidos se financia mayoritariamente a través de Medicaid (30), que aporta aproximadamente 0,34% del PIB (19). El otro 0,06% que completa el presupuesto proviene principalmente de programas locales (31).

En el modelo australiano, el presupuesto se compone de la siguiente manera: 75,4% de impuestos generales, recaudados por gobierno central; 20,7% de copagos; 3,8% de otras fuentes como seguros privados y 1,1% correspondiente a impuestos generales de gobiernos regionales (25).

Los modelos más generosos son los de Dinamarca y Japón. El primero tiene una base amplia de contribuciones a través de los municipios y subvenciones de impuestos generales del gobierno central (28). El modelo japonés se financia mediante impuestos generales, regionales, locales, seguros, y el cobro de copagos. Los impuestos generales constituyen 50% del presupuesto y se desgranan de la siguiente manera: 25% es aporte del gobierno central, 12% proviene de las prefecturas y 13% de las municipalidades. La otra mitad tiene dos fuentes: 22% es aportado por las cuotas de los asegurados primarios, y 28% por los asegurados secundarios (27). El seguro de cuidado a largo plazo se financia con la retención de 0,9% del salario de los trabajadores de 40 y más años. El copago se estructura por tramos de ingresos. Hasta el 2015, el copago fue de 10% para todos los usuarios no pobres, que están exentos. Ese año aumentó a 20% para aquellos con ingresos anuales superiores a US$25 613 (dólares estadounidenses). En el 2018 aumentó a 30% para los de ingresos mayores a US$32 712 (32).

**CUADRO 4.** Comparación de costos y esquemas de financiamiento de los programas de cuidados de larga duración de Costa Rica y otros países

**Países**	**Inversión pública** **(% del PIB)**	**Fuentes de financiamiento público**	**Copago** **(Sí/No)**
Costa Rica	Aspira a 0,8	Impuestos generales	Sí
Uruguay	<0,1	Impuestos generales	Sí
Dinamarca	2,5	Impuestos generales y municipales	Sí
Japón	2,07	Impuestos generales, regionales y municipales Seguros con impuestos a la nómina	Sí
España	0,77	Impuestos generales, regionales	Sí
Estados Unidos de América	0,4	Impuestos generales y regionales	Sí
Australia	1	Impuestos generales y regionales	Sí

PIB, producto interno bruto.

***Fuente:*** elaboración propia.

## DISCUSIÓN

Como consecuencia del envejecimiento demográfico, hace cuatro décadas los países de altos ingresos iniciaron su desarrollo de CLD (6). En las Américas, este proceso apenas comienza. Aunque existen actividades relacionadas en países como Brasil y Chile (33), solo hay dos modelos nacionales hoy en día: el de Uruguay, que inició en el 2015, y el de Costa Rica, que comenzó en el 2021 (3,8).

Al comparar el diseño del SNC con la evidencia internacional, se identificaron enseñanzas útiles para el desarrollo de otros CLD en la Región.

En cuanto al acceso, los modelos tienden al universalismo, aunque algunos consideran umbrales de edad, como Australia, Japón y Uruguay (11,27,14). Otros modelos no lo hacen (8-10).

En la mayoría de los casos estudiados, los sistemas se financian con impuestos generales, regionales, seguros y copagos (5). Costa Rica coincide parcialmente en esta estructura con impuestos generales y copagos pero, al igual que España y Uruguay, no incluye seguros como Japón (32). Además, no incluye financiamiento de entidades subnacionales como los modelos más abarcadores (12,17,19,34). La falta de nuevos recursos puede implicar dificultades en su desarrollo, como en Uruguay (14).

Los costos de los programas de CLD suelen ser elevados. Se ubican entre 2,5% del PIB en Dinamarca (26) y 0,4% del PIB en Estados Unidos (19). La excepción es Uruguay, que no supera el 0,1% del PIB (14). Costa Rica tiene un esquema de financiamiento similar al uruguayo. No acompaña los programas de CLD con nueva financiación específica, y de momento, se limita a reasignaciones presupuestarias (8).

La cobertura de servicios es variable. Mientras Australia cubre a 31,5% de los mayores de 65 y más años, Uruguay cubre a menos de 1% (15,22). Costa Rica prevé cubrir a 20% de mayores (8). Los países más envejecidos no tienen la mayor cobertura (10,18).

En cuanto a los tipos de servicios, aunque predomina la orientación a la atención domiciliaria, la gama es heterogénea. Se destaca la variedad de servicios en Australia, Dinamarca y Japón (15,9,23), que incluyen servicios de respiro y otros complementarios. Uruguay no incluye atención residencial en su sistema, lo hace solo para temas de supervisión y acreditación (13). Esto no ocurre en otros modelos. El SNC incluye telesalud y plataformas tecnológicas de integración de registros de sistemas de salud y de CLD. La oferta de servicios es similar al modelo español y uruguayo e incluye, además, servicios de respiro como Australia.

La mayoría de los modelos incluyen el servicio de transferencias monetarias (35); sin embargo, su desarrollo ha sido poco favorable, tal como lo ilustra el SAAD que, luego de iniciar ampliamente con esta prestación, ahora prioriza concediendo servicios de base domiciliaria (10). Costa Rica lo incluye solo bajo condiciones particulares.

Este estudio tiene dos limitaciones. La primera es el contraste entre la escasez de la bibliografía científica sobre CLD publicada en América Latina y la abundancia de bibliografía institucional. La segunda es de orden metodológico, porque la bibliografía disponible impide incluir los gastos del bolsillo como eje de análisis para generar enseñanzas sobre CLD en las Américas.

## Conclusiones

Costa Rica atendió la realidad del envejecimiento acelerado y su nuevo programa de CLD incorpora las principales enseñanzas obtenidas de otras experiencias en diferentes países. Prioriza la atención domiciliaria, otorga cobertura a una proporción de adultos mayores similar a la media de otros modelos, desarrolla parámetros para medir la calidad, crea medios de capacitación para los cuidadores formales y servicios de respiro para los familiares.

No obstante, aunque planifica destinar un porcentaje del PIB similar al de España, su esquema de financiamiento es limitado, semejante al del modelo uruguayo. Esto hace que no cuente con recursos económicos suficientes para su aplicación en el corto plazo, lo que pone en riesgo el incremento de la cobertura y la sostenibilidad financiera. Además, todos los sistemas son creados por ley, a diferencia del SNC. Este escenario genera incertidumbre y lo supedita legalmente a las metas propuestas para el 2031.

## Recomendaciones

La mayor expansión pública de programas de CLD en países de ingresos medianos será en las Américas. Atender esta realidad es posible y, Costa Rica es el primer país de ingresos medianos de la Región que avanza en este sentido. Su experiencia provee enseñanzas para el desarrollo de legislación sobre nuevos modelos que prioricen la atención domiciliaria, se formalicen programas de capacitación para cuidadores, se creen servicios de respiro, se limiten las transferencias monetarias, se establezcan parámetros de calidad y se incorporen esquemas de financiamiento sostenibles con una amplia y generosa base fiscal.

## Declaración.

Las opiniones expresadas en este manuscrito son únicamente responsabilidad de los autores y no reflejan necesariamente los criterios ni la política de la RPSP/PAJPH y/o de la Organización Panamericana de la Salud.
